# Exploring the Influence of Signal Molecules on Marine Biofilms Development

**DOI:** 10.3389/fmicb.2020.571400

**Published:** 2020-11-13

**Authors:** Ruojun Wang, Wei Ding, Lexin Long, Yi Lan, Haoya Tong, Subhasish Saha, Yue Him Wong, Jin Sun, Yongxin Li, Weipeng Zhang, Pei-Yuan Qian

**Affiliations:** ^1^Department of Ocean Science, Hong Kong University of Science and Technology, Kowloon, Hong Kong; ^2^Hong Kong Branch of Southern Marine Science and Engineering Guangdong Laboratory (Guangzhou), Hong Kong University of Science and Technology, Kowloon, Hong Kong; ^3^College of Marine Life Sciences, Ocean University of China, Qingdao, China; ^4^Institute for Advanced Study, Shenzhen University, Shenzhen, China; ^5^The Swire Institute of Marine Science, The University of Hong Kong, Pokfulam, Hong Kong; ^6^The Department of Chemistry, The University of Hong Kong, Pokfulam, Hong Kong

**Keywords:** marine biofilms, signal molecules, PQS, Erythrobacter, marine bacteria

## Abstract

Microbes respond to environmental stimuli through complicated signal transduction systems. In microbial biofilms, because of complex multiple species interactions, signals transduction systems are of an even higher complexity. Here, we performed a signal-molecule-treatment experiment to study the role of different signal molecules, including N-hexanoyl-L-homoserine lactone (C6-HSL), N-dodecanoyl-L-homoserine lactone (C12-HSL), *Pseudomonas* quinolone signal (PQS), and cyclic di-GMP (c-di-GMP), in the development of marine biofilms. Comparative metagenomics suggested a distinctive influence of these molecules on the microbial structure and function of multi-species biofilm communities in its developing stage. The PQS-treated biofilms shared the least similarity with the control and initial biofilms. The role of PQS in biofilm development was further explored experimentally with the strain *Erythrobacter* sp. HKB8 isolated from marine biofilms. Comparative transcriptomic analysis showed that 314 genes, such as those related to signal transduction and biofilm formation, were differentially expressed in the untreated and PQS-treated *Erythrobacter* sp. HKB8 biofilms. Our study demonstrated the different roles of signal molecules in marine biofilm development. In particular, the PQS-based signal transduction system, which is frequently detected in marine biofilms, may play an important role in regulating microbe-microbe interactions and the assemblage of biofilm communities.

## Introduction

Prokaryotic microbes respond to various environmental stimuli by employing signal transduction systems (Gotoh et al., 2010). Signal transduction refers to the process of identifying and converting external signals into gene activation and cellular responses to unambiguous environmental signals. Many different signal molecules are used by microbes (bacteria and archaea) to respond to environmental stimuli and to communicate with one another (Hoch, 2000; Stock et al., 2000; Laub and Goulian, 2007; Bourret and Silversmith, 2010; Gotoh et al., 2010). For example, many proteobacteria use acyl-homoserine lactones (AHLs) to regulate diverse physiological processes and group activities, including swarming motility, antibiotic resistance, virulence, conjugal plasmid transfer, sporulation, and biofilm formation (Miller and Bassler, 2001; Waters and Bassler, 2005; Bassler and Losick, 2006). Different kinds of AHLs were detected in subtidal marine biofilms, including C6∼C14-HSL and 3-oxo-C4∼C14-HSL, suggesting that the AHL-producing bacterial community could be as dynamic as the microbial community during subtidal biofilm formation (Huang et al., 2009). Moreover, *Pseudomonas* strains are common strains in marine biofilms, and they produce the *Pseudomonas* quinolone signal (PQS), which could act as a signal molecule that affects bacterial interspecies behavior and communication in both Gram-positive and Gram-negative bacteria, including *Escherichia coli* NCIMB11943, *Vibrio fischeri* ES114, *Proteus vulgaris* NCIMB12426, and *Bacillus subtilis* NCTC10073 (Mooij et al., 2011). For example, PQS regulates membrane vesicle formation in *E. coli* and *B. subtilis*, indicating that the membrane structure of bacteria may not be a limiting determinant for interactions involving PQS (Tashiro et al., 2010; Mooij et al., 2011), and considerable promotion in biofilm formation of a marine-derived extremophile *Halanaerobium* spp. could be achieved by PQS (Monzon et al., 2016). In addition, a marine bacterium *Pelagibaca bermudensis* releases PQS and its precursor 2-heptyl-4-quinolone (HHQ) under varying environmental conditions (Patidar et al., 2017). Cyclic di-GMP (c-di-GMP) is produced as a ubiquitous second messenger to control cell adhesion and persistence of multicellular communities (Jenal and Malone, 2006; Valentini and Filloux, 2016). However, despite these earlier studies, the effect of signal transduction on natural marine biofilms remains unresolved.

As an important microbial lifestyle, biofilms are complex and dynamic surface-associated communities that are mostly formed by multiple microbial species (Abraham, 2016; Flemming et al., 2016). Many previous studies have used single-species biofilms and molecular tools to study the molecular and physiological mechanisms of biofilm development. *Pseudomonas aeruginosa* is one of the species models initially used in studies investigating the development of biofilms. For example, the *las* signal transduction system was report to be important in the creation of mature *P. aeruginosa* biofilms (Davies et al., 1998), and one of first investigations of genes implicated in *P. aeruginosa* biofilm initiation suggested that flagella were a crucial part in the assemblage of biofilms (O’Toole and Kolter, 1998). *Bacillus subtilis* has long been studied as another robust model organism, and its biofilm development is found to be regulated through several integrated signaling pathways (Beauregard et al., 2013; Mielich-Süss and Lopez, 2015). These studies of biofilms using model bacterial species mainly relied on molecular and biochemical tools. For example, the signal molecules produced by cultured bacteria could be identified easily using liquid chromatography-mass spectrometry (Ortori et al., 2014).

However, the significant progress made in high throughput sequencing and bioinformatic analysis over the past few years offers a unique opportunity to evaluate the roles and functions of complex microbial communities and to enhance our knowledge in ecologically relevant field studies, including marine biofilms (Antunes et al., 2019). For instance, a metagenomic study of the biofilm communities from the Lost City hydrothermal field has identified abundant transposases, highlighting the significance of lateral gene transfer in extreme environments (Brazelton and Baross, 2009). Mosier et al. (2015) reported a model biofilm-based ecosystem, in which elevated temperature had a physiological influence on specific microbial groups. In a previous study, we investigated the microbial community structures of subtidal biofilms in different ages; the results showed that biofilms older than 12 days were similar to one another in terms of microbial composition but were different from biofilms younger than 9 days (Chung et al., 2010). Therefore, high throughput sequencing and bioinformatic analysis grew into a powerful tool for the study of natural biofilm communities.

In the present study, we hypothesized that particular signal molecules play important roles in shaping the microbial communities in biofilms. To test this hypothesis, we developed biofilms in the coastal marine subtidal zone of the Hong Kong coast and treated them with different signal molecules. The effects of various signal molecules on the taxonomic structure and functional profiles of biofilms were estimated by metagenomic analyses. Transcriptomic analysis was performed then to confirm the role of a signal molecule in regulating the gene expression of a marine-derived bacterium.

## Materials and Methods

### Signal Molecules Treatment Experiment of Biofilms Developed in a Subtidal Zone

Biofilms used for the signal molecules treatment experiment were developed on plastic Petri dishes deployed in Port Shelter, Hong Kong (22°33'82 N, 114°26'85 E) in January 2018. Biofilms that had been growing for 9 days at a depth of 2 meters below the surface were collected and transported to the laboratory immediately. Meanwhile, 2 L of seawater collected from the adjacent water column was transported to the laboratory and filtered through 0.1 μm membranes (Millipore, Massachusetts, United States). The Petri dishes with the 9-day-old biofilms were randomly divided into 11 groups (each containing 10 Petri dishes): two groups were treated with C6-HSL, two groups were treated with C12-HSL, two groups were treated with c-di-GMP, two groups were treated with PQS, two groups were placed in seawater without any molecule treatment and defined as the “control” biofilms, and one group was immediately subjected to DNA extraction without any sort of treatment and defined as the “initial” biofilm. According to previous studies, the final concentrations of the C6-HSL, C12-HSL, c-di-GMP, and PQS molecules used in the treatments were 10, 10, 2.5, and 10 μM, respectively (Lynch et al., 2002; Hengge, 2009). After adding the molecules, the biofilms were exposed to the relevant treatments for 24 h at room temperature. Biofilms were removed from Petri dishes using sterile cotton tips, and the tips were washed by DNA storage buffer (500 mM NaCl, 50 mM Tris-HCl, 40 mM EDTA, and 50 mM glucose) and then biofilms were collected by centrifugation at 4000 × *g* for 10 min before DNA extraction and metagenomic sequencing.

### DNA Extraction, Metagenomic Sequencing, and Analyses

DNA extraction was performed using a microbial genomic DNA extraction kit (TIANGEN Biotech, Beijing, China). Metagenomic sequencing was performed on an Illumina HiSeq X Ten platform at Novogene (Beijing, China). All Illumina pair-end raw reads (2 × 150 bp) were qualified using the next-generation sequencing (NGS) quality control (QC) toolkit (version 2.0; Patel and Jain, 2012). Low-quality reads with quality scores less than 20 for more than 30% of the read length were removed. All the metagenomes were normalized to 10,000,000 reads per sample. Metagenome information of the biofilms treated with signal molecules is given in Supplementary Table S1, and the accession number is Bioproject PRJNA513416. The details and significance of signal molecules in this experiment are shown in Supplementary Table S2.

The taxonomy diversity of prokaryotes at phylum and genus levels was identified using Parallel-Meta 3 V.3.4.4 (Jing et al., 2017). Briefly, in this software, 16S rRNA gene sequences are extracted from the metagenomes based on Hidden Markov Models using HMMER (version 3.1, *e*-value <1e-5; Eddy, 2011). 16S rRNA gene sequences are the mapped by Bowtie2 (version 2.0.0; Langmead and Salzberg, 2012) to a modified GreenGenes 13_8 database (DeSantis et al., 2006) which had been further annotated using the RDP (Cole et al., 2009), and SILVA (Quast et al., 2012) and is distributed with of Parallel-Meta 3 V.3.4.4 (Jing et al., 2017). According to taxonomic structures, cluster analysis was established through unweighted pair-group method with arithmetic means algorithm (UPGMA) and used Bray-Curtis distances. One-way permutational multivariate analysis of variance (oneway-PERMANOVA) and SIMPER analysis were performed using the software PAST (version 2.0; Hammer et al., 2001) based on Bray-Curtis distances. Alpha-diversity was estimated based on the Shannon index of genus taxa and visualized by GraphPad Prism version 8.0.2 (GraphPad Software, CA, United States).

To draw the functional profile of the molecule-treated biofilms, all the metagenomes reads were mapped to the eggNOG database (version 4.5; Huerta-Cepas et al., 2016) using DIAMOND BLASTx (*e*-value <1e-7; >60% sequence identity for >60% of the read length; Buchfink et al., 2015) and annotated by the SEED database (level 1) in MEGAN (version 6.9.3; Huson et al., 2016). The relative abundance of a given functional category in each sample was calculated by the percentage of metagenomic reads mapped to genes belonging to that category. Moreover, to identify the taxonomic affiliation of the signal transduction genes, the reads mapped to the regulation and cell signaling category (level 2, SEED subcategory) were selected to perform DIAMOND BLASTx against the NCBI *nr* database. Subsequently, the results were classified into phylum and genus levels in MEGAN. Statistical analyses were performed by Student’s *t*-test and *U*-test in the software STAMP (version 2.1.3; Parks et al., 2014).

### Bacterial Isolation From Marine Biofilms

Bacterial isolation from marine biofilms developed on the plastic Petri dishes followed the procedure described by Ding et al. (2019). Briefly, the biofilms were removed from Petri dishes using sterile cotton tips, diluted 10 or 100 times by Marine Broth 2216E (BD Franklin Lakes, NJ, United States), spread on Marine Broth Agar plates (BD Difco 2216, New Providence, NJ, United States), and incubated at 22°C for 24 h. Colonies were examined under a dissecting microscope for morphological characteristics. Then, conspicuous colony types were isolated, and their 16S rRNA genes were amplified using the 16F/1492R primers and sequenced using the Sanger method at BGI (Beijing, China) to identify the taxonomy of the isolates.

An isolated strain of Alphaproteobacteria *Erythrobacter* sp. HKB8 was selected for the treatment experiment involving PQS. Genomic DNA of this bacterium was extracted using a microbial genomic DNA extraction kit (TIANGEN Biotech, Beijing, China) and sequenced on the Illumina HiSeq X Ten platform at Novogene (Beijing, China). Genome assembly was performed using the software SPAdes (version 3.11.1) following the quality control of raw reads (Bankevich et al., 2012), and then the completeness of genome was estimated by CheckM (lineage_wf; Parks et al., 2015). Open reading frames (ORFs) were predicted using Prodigal (Hyatt et al., 2010). The ORFs were annotated using BLASTp (*e*-value <1e-7; >60% sequence identity for >60% of the read length) against the Kyoto Encyclopedia of Genes and Genomes (KEGG; Kanehisa et al., 2012). The genome information of *Erythrobacter* sp. HKB8 is given in Supplementary Table S3, and the accession number is Bioproject PRJNA513246.

### Bacterial Growth Monitoring and Biofilm Formation Assay of *Erythrobacter* sp. HKB8

Growth curves of *Erythrobacter* sp. HKB8 were performed in triplicated 2 ml using Marine Broth 2216E (BD Franklin Lakes, NJ, United States) with or without 10 μM PQS at 22°C under 250 rpm. The optical density of bacterial cells at 595 nm was measured after 2, 4, 6, 8, 12, 16, 20, 24, 30, 36, 42, 48, 54, 60, and 66 h using the SmartSpec Plus Spectrophotometer of Bio Rad Laboratories (Hercules, CA, United States). After 20 h (OD = 0.5), the bacterial culture without PQS added was transferred to 96-well microplates for biofilm formation assay. PQS (10 μM) was added to the experimental group in 10 replicates along with 10 controls. The bacterial cells treated or not with PQS in microplates were incubated at 22°C. After 24 h, the supernatants and loosely attached bacterial cells were washed thrice with 200 μl of phosphate buffer solution (PBS). The biomass of the formed bacterial biofilm was measured using a Microplate Spectrophotometer (Thermo Fisher Scientific) based on the crystal violet method (Müsken et al., 2010). The results were visualized in GraphPad Prism version 8.0.2 (GraphPad Software, CA, United States).

### PQS Treatment Experiment and Transcriptomic Analysis of *Erythrobacter* sp. HKB8 Biofilms

*Erythrobacter* sp. HKB8 was cultured in Marine Broth 2216E (BD Franklin Lakes, NJ, United States) under 250 rpm at 22°C. After 16 h, when *Erythrobacter* sp. HKB8 was in the logarithmic growth phase (OD_595_ = 0.4), 6 ml bacterial culture was added to the wells of six-well plates to allow biofilm formation. After 3 h, 10 μM PQS was added to the wells, and the cultures were incubated for 24 h. After the incubation, the planktonic phase was removed and the cells that formed the biofilms were removed by sterile cotton tips and washed down by RNAprotect Bacteria Reagent (Qiagen, Hilden, Germany). In parallel, the biofilms of *Erythrobacter* sp. HKB8 were developed in wells that were left untreated with PQS and served as the control. Three replicates were generated for each treatment. RNA extraction was performed at Novogene (Beijing, China), where Ribo-Zero strand-specific libraries were constructed after the reduction of rRNA. The libraries were sequenced on the HiSeq X Ten System, and then all Illumina pair-end raw reads (2 × 150 bp) were qualified using the NGS QC toolkit (version 2.0; Patel and Jain, 2012).

After passing quality control, the transcriptomic reads were mapped to the ORFs using Bowtie2 (version 2.0.0; Langmead and Salzberg, 2012). The coverage was calculated using SAMtools (Li et al., 2009) to generate the gene expression profiles, which were displayed in transcripts per million (TPM). Shapiro-Wilk and Levene’s tests were performed to examine the normality and homogeneity of variances, respectively. Two-tailed Student’s *t*-test was performed to compare the PQS-treated and control biofilm cultures. The results were visualized with a volcano plot generated in Excel and with a heatmap generated by Cluster 3.0 (De Hoon et al., 2004) and Java Treeview (Saldanha, 2004). The detailed transcriptome information of *Erythrobacter* sp. HKB8 is presented in the Supplementary Table S4, and the accession number is Bioproject PRJNA513396.

## Results

### Influence of Signal Molecules on the Taxonomic Composition of Biofilm Communities

**Figure 1:**
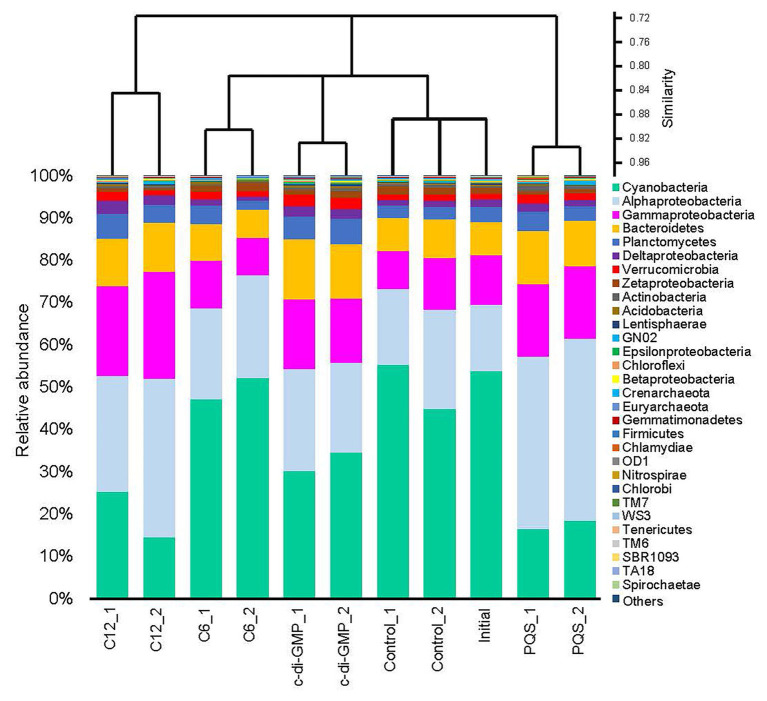
Phylum-level composition of the biofilms in the signal molecules treatment experiment. Biofilms that had been growing for 9 days in the subtidal zone of Hong Kong waters were collected and subjected to different signal molecules treatments in the laboratory. Then, the genomic DNA of each biofilm sample was sequenced for metagenomic analysis. The biofilms treated with seawater were labeled as “control,” and the untreated biofilm was labeled as “initial.” Except for the “initial” biofilm, each treatment had two biological replicates. Proteobacteria were classified down to the class level. The 30 most abundant taxa (ranked by maximum abundance) are shown, and the other taxa are included under the classification of “Others.”

To test our hypothesis that signal molecules had influence on biofilm communities, N-hexanoyl-L-homoserine lactone (C6-HSL), N-dodecanoyl-L-homoserine lactone (C12-HSL), c-di-GMP, and PQS were added to biofilms pre-established *in situ* on plastic Petri dishes. Biofilms developed in the subtidal zone for 9 days (the initial sample) were collected and incubated with different signal molecules for 24 h. Community compositions were compared between the molecule-treated (the treated group) and untreated biofilms (the control group). We generated a dataset that was 197.13 Gb in size (Supplementary Table S1) by sequencing 11 metagenomes. Taxonomic analysis based on gene sequences extracted from the metagenomes revealed 30 phyla or Proteobacterial classes of microbes with the highest abundance (Figure 1). In most of the samples, Cyanobacteria showed the highest abundance, followed by Alphaproteobacteria and Gammaproteobacteria. Cluster analysis based on the relative abundance of phyla or Proteobacterial classes clustered replicates that shared more than 85% similarity, but separated samples treated with different molecules, and the initial and control groups, indicating that each molecule had a specific impact on the structure of microbial communities in the biofilms (Figure 1). Without any treatment, the initial and control groups shared about 90% similarity, thereby reflecting the minor influence of a 24 h *ex situ* incubation in seawater on marine biofilms (Figure 1). Although both C6-HSL and C12-HSL are AHLs signals, the similarity of microbial composition between the C12-HSL-treated and the control biofilms was lower than that between the C6-HSL-treated biofilms and the control. The biofilms treated with PQS and C12-HSL showed the least similarity to the control and initial biofilms (Figure 1).

The 16S rRNA gene sequences extracted from the metagenomes were further classified at the genus level, indicating the significant differences among groups (PERMANOVA, *p* < 0.01). Among 119 classified genera, the 30 most abundant genera are illustrated in Supplementary Figure S1. Up to 65% of the 16S rRNA gene sequences could not be classified at the genus level, suggesting that a large proportion of unclassified microbes existed in marine biofilms. An unidentified genus in Rhodobacteraceae was the most abundant genus in all the samples, followed by *Alteromonas* and an unclassified genus in Hyphomonadaceae, *Sulfitobacter*, and *Mariprofundus* (Supplementary Figure S1). Cluster analysis suggested that the PQS-treated and the C12-HSL-treated biofilms were the most distant from the control and initial groups in terms of taxonomic structure (Supplementary Figure S1). In addition, alpha-diversity based on the taxonomic classification at genus level confirmed the changes of microbial diversity between the control and treated biofilms. The Shannon diversity index in PQS-treated biofilms was significantly higher than that in the control biofilms (two-tailed Student’s *t*-test, *p* < 0.05), whereas no significant changes were found in other signal groups (Supplementary Figure S2). Results of SIMPER analysis revealed that the unclassified genera contributed to the 49.8% difference between the PQS-treated biofilms and the control biofilms. The top 30 contributing genera (total contribution of 36.27% difference) are displayed in Supplementary Figure S3. Based on the relative abundance, most of these genera were proportionally overrepresented in the PQS-treated biofilms, such as *Sulfitobacter*, *Alteromonas*, *Thalassospira*, *Ruegeria*, and *Erythrobacter* (Supplementary Figure S3).

### Influence of Signal Molecules on the Functional Profile of Biofilm Communities

**Figure 2:**
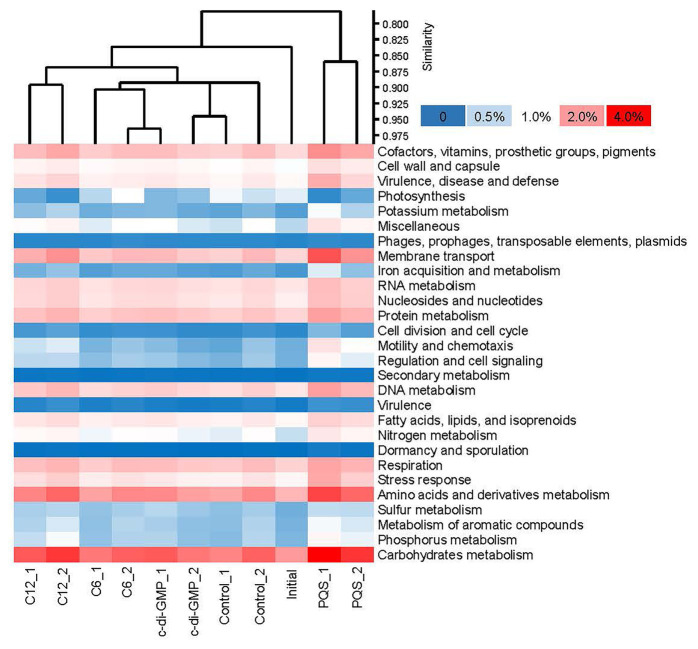
Functional profiles (SEED level 1) of the multi-species biofilms in the signal molecules treatment experiment. The profiles were generated by searching the metagenomic reads against the eggNOG database and by classifying the functions according to the SEED category, and the relative abundance of each category was counted. Cluster analysis revealed that the *Pseudomonas* quinolone signal (PQS)-treated biofilms had the least similarity with the control and initial biofilms.

To improve our understanding of the effects of signal molecules on biofilm communities, the biofilm-derived gene sequences were classified into 28 functional categories when the functional profiles of the molecule-treated and control biofilms were compared at SEED level 1 (Figure 2). The result supported the argument that the signal molecules influenced the overall function of marine biofilms, and the biofilms treated with C12-HSL or PQS were located along two well-separated branches after a short treatment period (Figure 2). In details, the relative abundance (indicated by the percentage of mapped reads) of genes in the membrane transport category, the motility and chemotaxis category, and the regulation and cell signaling category were higher in C12-HSL or PQS groups than that in other biofilms, especially in the PQS-treated biofilms (Figure 2).

To further explore the enrichment of signal transduction genes after treatment with signal molecules, the taxonomic affiliations of the functional genes related to regulation and cell signaling in PQS-treated biofilms and in the control were established at the phylum and genus levels (Supplementary Figure S4). No significant difference was observed in terms of the composition of taxonomic affiliations (relative abundance of phyla or genera; *U*-test, *p* > 0.05), thereby manifesting that few taxa with signal transduction genes were specific to PQS treated or untreated biofilms. Surprisingly, when we searched the genera possessing signal transduction genes (showed in Supplementary Figure S4B) in our genus level classification of PQS-treated metagenomes (showed in Supplementary Figure S1), all common genera displayed higher relative abundance than in the controls, and 12 of these genera showed significantly higher concentration in PQS-treated biofilms (two-tailed Student’s *t*-test, *p* < 0.05; Supplementary Figure S5). In addition, 16 genera possessing signal transduction genes were found among the top 30 contributing genera (shown in Supplementary Figure S3), and these 16 genera occupied over 21% difference in the comparison of taxonomic composition between the PQS-treated biofilms and the control biofilms, such as *Alteromonas*, *Donghicola*, *Hyphomonas*, and *Erythrobacter* (Supplementary Figure S5).

### Influence of PQS on Biofilm Formation and Gene Expression in Biofilm Development of an *Erythrobacter* Strain Isolated From Marine Biofilms

Based on the influence of signal molecules on marine biofilm community, we hypothesized that specific microbes in biofilms could respond to signal molecules, possibly through non-universal signal receptor genes. The PQS and C12-HSL treatments displayed the most significant changes in the taxonomic structure of biofilms, whereas PQS had the strongest influence on the functional profiles in the signal molecules treatment experiment. Thus, we chose PQS for subsequent exploration. To further test the hypothesis, we isolated and sequenced tens of bacterial strains from marine biofilms and selected one strain from *Erythrobacter* (referred to as *Erythrobacter* sp. HKB8) for signal molecules treatment. This bacterium was selected because: (1) *Erythrobacter* was one of the top 30 genera that contributed to the difference between the PQS-treated biofilms and the control biofilms (Supplementary Figure S3); (2) the relative abundance of *Erythrobacter* in the PQS-treated biofilm communities was more than 2-fold higher than that in the control (two-tailed Student’s *t*-test, *p* < 0.05; Supplementary Figure S3); (3) signal transduction genes were present in *Erythrobacter* in PQS-treated natural biofilms (Supplementary Figure S4); and (4) *Erythrobacter* could be isolated from natural marine biofilms and is culturable in the laboratory. Thus, this bacterium could be a good object to investigate interspecies response to PQS. The 16S rRNA gene sequence analysis showed that this bacterium had 99% identity to *Eryhthrobacter formosense* sp. nov., a carotenoid-producing Alphaproteobacterium isolated from coastal seawater.

**Figure 3:**
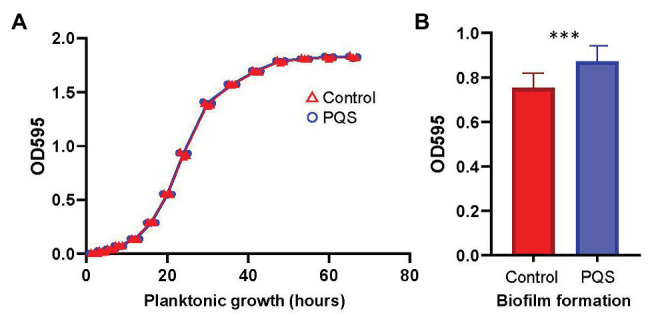
Influence of 10 μM PQS on planktonic growth and biofilm formation of *Erythrobacter* sp. HKB8. **(A)** Planktonic growth and **(B)** biofilm formation (^***^ *p* < 0.001).

The planktonic growth curves of *Erythrobacter* sp. HKB8 with or without PQS in marine broth at 22°C were drawn (Figure 3A). The two curves were nearly identical, suggesting that PQS has minimal impact on the growth of the *Erythrobacter* sp. HKB8 strain. In parallel, the biofilm of *Erythrobacter* sp. HKB8 cultured with PQS had a higher biomass than the one cultured without PQS (two-tailed Student’s *t*-test, *p* < 0.001; Figure 3B).

**Figure 4:**
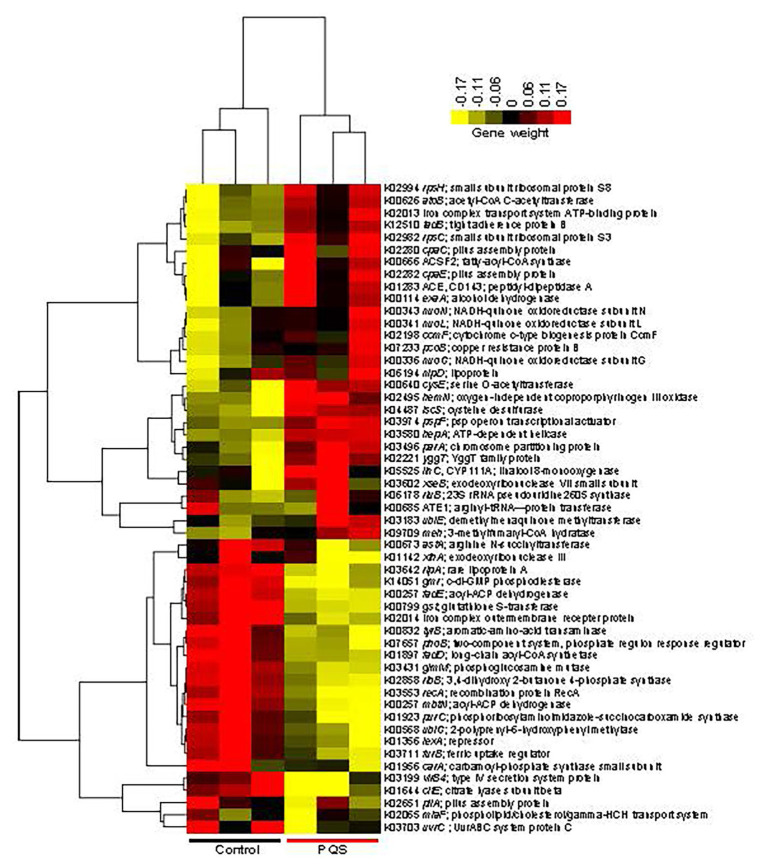
Heatmap showing the differentially expressed genes between the PQS-treated biofilms and the control biofilms of *Erythrobacter* sp. HKB8. A *p*-value cutoff of 0.01 in a two-tailed Student’s *t*-test was used to select the significantly changed genes. Then, the transcripts per million (TPMs) of genes that could be annotated by the Kyoto Encyclopedia of Genes and Genomes (KEGG) database were used to plot the heatmap.

Genome sequencing and annotation predicted 2,637 ORFs from the genome of *Erythrobacter* sp. HKB8, which was 99.48% complete (Supplementary Table S3). Comparative transcriptomic analysis showed that 314 genes were differentially expressed in the *Erythrobacter* sp. HKB8 biofilms treated with and without PQS (two-tailed Student’s *t*-test, *p* < 0.05): a total of 170 genes were upregulated in the PQS-treated biofilms, whereas the other 144 genes were upregulated in the control biofilms (Supplementary Figure S6). In detail, the genes related to PQS response and stress resistance, such as *nuoN* (NADH-quinone oxidoreductase subunit *N*), *ubiE* (demethylmenaquinone methyltransferase), and *pcoB* (copper resistance protein B), showed higher expression levels, whereas the genes related to other signal transduction systems, including *phoB* (two-component system; phosphate regulon response regulator) and *gmr* (c-di-GMP phosphodiesterase) showed lower expression levels after PQS treatment (two-tailed Student’s *t*-test, *p* < 0.01; Figure 4). Moreover, *tadB* (tight adherence protein B), which is related to biofilm formation, displayed higher expression levels after PQS treatment (two-tailed Student’s *t*-test, *p* < 0.01; Figure 4); this result was consistent with the biofilm formation assay outcome that *Erythrobacter* sp. HKB8 showed higher biomass when cultured with PQS (Figure 3B).

## Discussion

Signal transduction plays key roles in the biofilm development of single bacterial species (Beauregard et al., 2013; Mielich-Süss and Lopez, 2015). Signal transduction genes are important in various bacterial physiological activities, such as symbiosis, virulence, competence, conjugation, antibiotic production, motility, sporulation, and biofilm formation (Fineran et al., 2005; Bassler and Losick, 2006; Jitacksorn and Sadowsky, 2008; Antunes et al., 2010). Regarding multi-species biofilms in natural environments, analysis of the chemical profiles of subtidal biofilms revealed the dynamics of signal molecules associated with biofilm development (Huang et al., 2009). However, the potential effect of these changing molecules on the succession of bacterial community has not been clarified. According to our previous study, 9-day-old marine biofilms were in the developing stage with high biodiversity (Chung et al., 2010), thereby they were adequate for our experiments. In present study, the results of signal molecules treatment experiment with 9-day-old biofilms supported different functions of signal molecules in molding the development of marine biofilms.

Marine biofilms are considered as a bank and reservoir of hidden microbial diversity, and global subtidal biofilms displayed high similarity in terms of taxonomic and functional patterns (Zhang et al., 2019). Our results indicated that each signal molecule could change the composition of marine biofilms by affecting the relative abundance of certain microbial taxa, resulting in the different signals with varying impacts on biofilm. C12-HSL had a stronger influence on the taxonomic and functional composition of biofilms than C6-HSL, and PQS shared the least similarity with the control group in terms of the functional profile, suggesting that some signal molecules could be involved in more interspecies interactions than others, even if some molecules belong to the same class of signal molecules (e.g., C6-HSL and C12-HSL belong to AHLs). A change in the abundance of several functional gene categories was observed in the metagenomic results of the signal molecules treated groups. Increased membrane transport genes supported the previous results that PQS is an integral membrane component and associated with the emergence of outer membrane vesicles, which promotes membrane curvature (Mashburn and Whiteley, 2005; Mashburn-Warren et al., 2008). Meanwhile, the genes related to signal transduction increases in relative abundance after signal molecules treatment whereas the taxonomic affiliation of these genes was not significantly changed. However, the relative abundance of the genera possessing signal transduction genes increased, which contributed much to the difference between the PQS-treated and control biofilms. Therefore, we inferred that during establishment and colonization of biofilms from a planktonic cells, when signal molecules are released, bacteria with interrelated signal transduction genes may be selected and enriched in biofilms, resulting in the accumulation and observed higher relative abundance of corresponding signal transduction genes, which indicates that signal transduction could be one of functional bases during biofilm development and assembly from a natural multi-species perspective. Nevertheless, since our results just showed the influence of signal molecules on natural marine biofilms in the developing stage, their effect on the initial stage of biofilm assemblage needs to be further explored in the future.

The results of the signal molecules treatment experiment and the biofilm formation assay of a single bacterial strain further highlighted how a signal molecule performs its role in biofilm assembly. PQS not only affects cellular respiration activity in *P. aeruginosa* but also acts as a signal molecule in multi-species community (Toyofuku et al., 2009; Mooij et al., 2011). *Erythrobacter* sp. HKB8, an abundant strain in marine biofilms, is a Gram-negative and rod-shaped bacterium frequently found in marine biofilms (Jones et al., 2007; Chen et al., 2013). Since PQS could repress the growth of several species including both Gram-negative and Gram-positive bacteria as an antibiotic, we tested the effect of PQS on the growth of our selected bacterium to suggest that PQS did not function as an antibiotic in this concentration of the study. On the contrary, biofilm formation of *Erythrobacter* sp. HKB8 was enhanced by PQS. Given that *Erythrobacter* belongs to Alphaproteobacteria, whereas *Pseudomonas* belongs to Gammaproteobacteria, PQS likely mediated interactions with microbes that are phylogenetically distant from one another. Our result is consistent with previous findings that 10 μM PQS did not suppress the growth of a *B. subtilis* marine isolate (Reen et al., 2012) and an *Algoriphagus* marine isolate (Mooij et al., 2011), and increased the biofilm formation of marine-derived *B. subtilis* and *Halanaerobium* spp. (Mooij et al., 2011; Reen et al., 2012; Monzon et al., 2016). This finding reminds us that since these antibiotic molecules are typically used for therapeutic purpose in our daily lives, the potential of the so-called antibiotics to act as signal molecules in natural conditions has been often ignored (Yim et al., 2007). Consistently, the transcriptomic analysis supported the influence of PQS on the expression of signal transduction genes in *Erythrobacter* sp. HKB8 biofilms. Compared with the control group, the expression of stress response genes in the PQS-treated biofilms was enhanced, suggesting that PQS may strengthen bacterial fitness in a given environment by increasing stress resistance (Lin et al., 2018). Interestingly, the genes related to PQS response were upregulated, whereas the expressions of other signal transduction systems were downregulated (e.g., c-di-GMP system and two-component systems), suggesting that PQS plays a role in signal transduction during biofilm formation, and it could affect gene expression through a relative dependent pathway apart from other signals instead of a uniform one. This phenomenon may be one of reasons why different microbial compositions were observed when the biofilm was treated by different signal molecules.

Since the interactions between chemical molecules and bacteria are highly complex in natural environments (Hosni et al., 2011; Whiteley et al., 2017), there are several limitations in the present study. On the one hand, signal transduction is not the only role of signal molecules in some prokaryotes. For example, a terrestrial bacteria *Erwinia carotovora* could utilize AHL-dependent sensing to regulate the exoenzyme and toxin production (Bainton et al., 1992), and a Gram-positive water bacterium *Ralstonia* sp. could employ its extracellular AHL-acylases (AhlM) that break the amide bond of AHLs as a nutrient to produce bacteria (Lin et al., 2003). On the other hand, bacteria are not the only actors involved in signal transduction in natural environments. For example, fungi are important members of marine biofilms, and they could produce metabolites to interfere with bacterial autoinducer-2 signal transduction, which is a signaling system implicated in the colonization of virulence and host (Lami et al., 2019); AHLs produced by the biofilms could attract the zoospores and induce calcium influx related to the regulation of flagellar movement, resulting in a decrease in swimming rate (Joint et al., 2007). Thus, further effort is needed to decipher the underlying mechanisms of interspecies interactions mediated by signal molecules in complicated environments.

To summarize, our signal molecules treatment experiment suggested the distinctive influence of signal molecules on the microbial structure and function of multi-species biofilm communities. More specifically, we concluded that the PQS signal transduction system, which is frequently detected in natural marine biofilms, played distinguishing roles in regulating microbe-microbe interactions and the assemblage of surface-associated microorganisms, implying the huge ecological potential of small signal molecules with low concentrations in natural biofilm ecosystems.

## Data Availability Statement

The datasets presented in this study can be found in online repositories. The names of the repository/repositories and accession number(s) can be found in the article/Supplementary Material.

## Author Contributions

P-YQ, WZ, and YL designed this study and wrote the related grant proposals. RW, WD, and YHW collected samples. RW and WD performed experiments and analyzed data. LL, YL, HT, SS, and JS provided biochemical and bioinformatic technical support. RW and WZ drafted the manuscript with input from every co-author. All authors contributed to the article and approved the submitted version.

### Conflict of Interest

The authors declare that the research was conducted in the absence of any commercial or financial relationships that could be construed as a potential conflict of interest.
