# Supplementary material for: Exploring the Influence of Signal Molecules on Marine Biofilms Development
Source: Front Microbiol. 2020 Nov 13;11:571400. doi: 10.3389/fmicb.2020.571400 (PMC7691533; doi:10.3389/fmicb.2020.571400)
Supplement: Supplementary file 1 [file Data_Sheet_1.pdf]

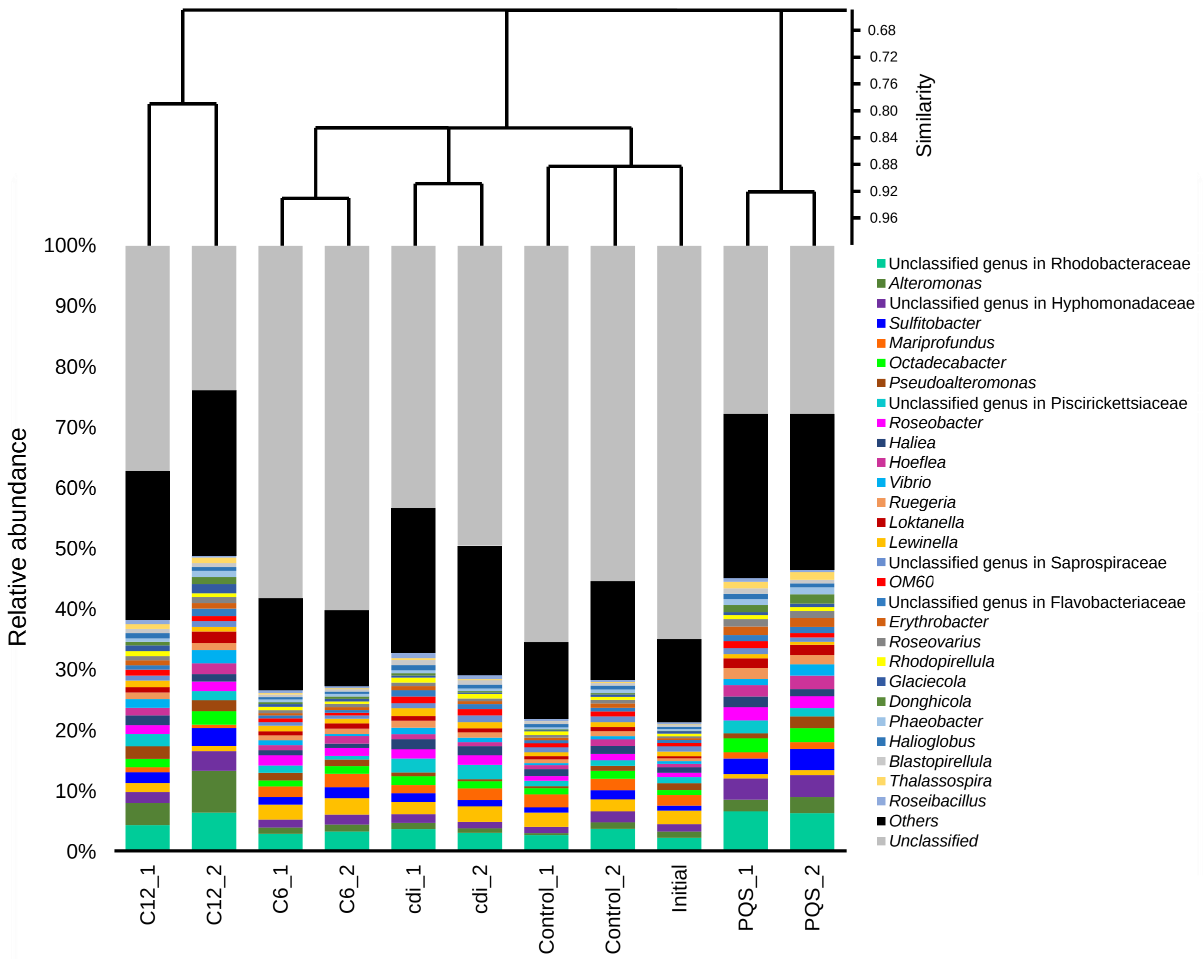

Supplementary Figure S1. Taxonomic compositions of the biofilms treated with signal molecules at the genus level. The biofilms treated with seawater are labeled as 'control', and the nine-day-old untreated biofilms are labeled as 'initial'. Except for the 'initial' biofilms, each treatment had two biological replicates. Taxonomic analysis was performed using the 16S rRNA gene sequences extracted from the metagenomes. The 30 most abundant taxa (including 'Unclassified') are given and the others are grouped into 'Others'.

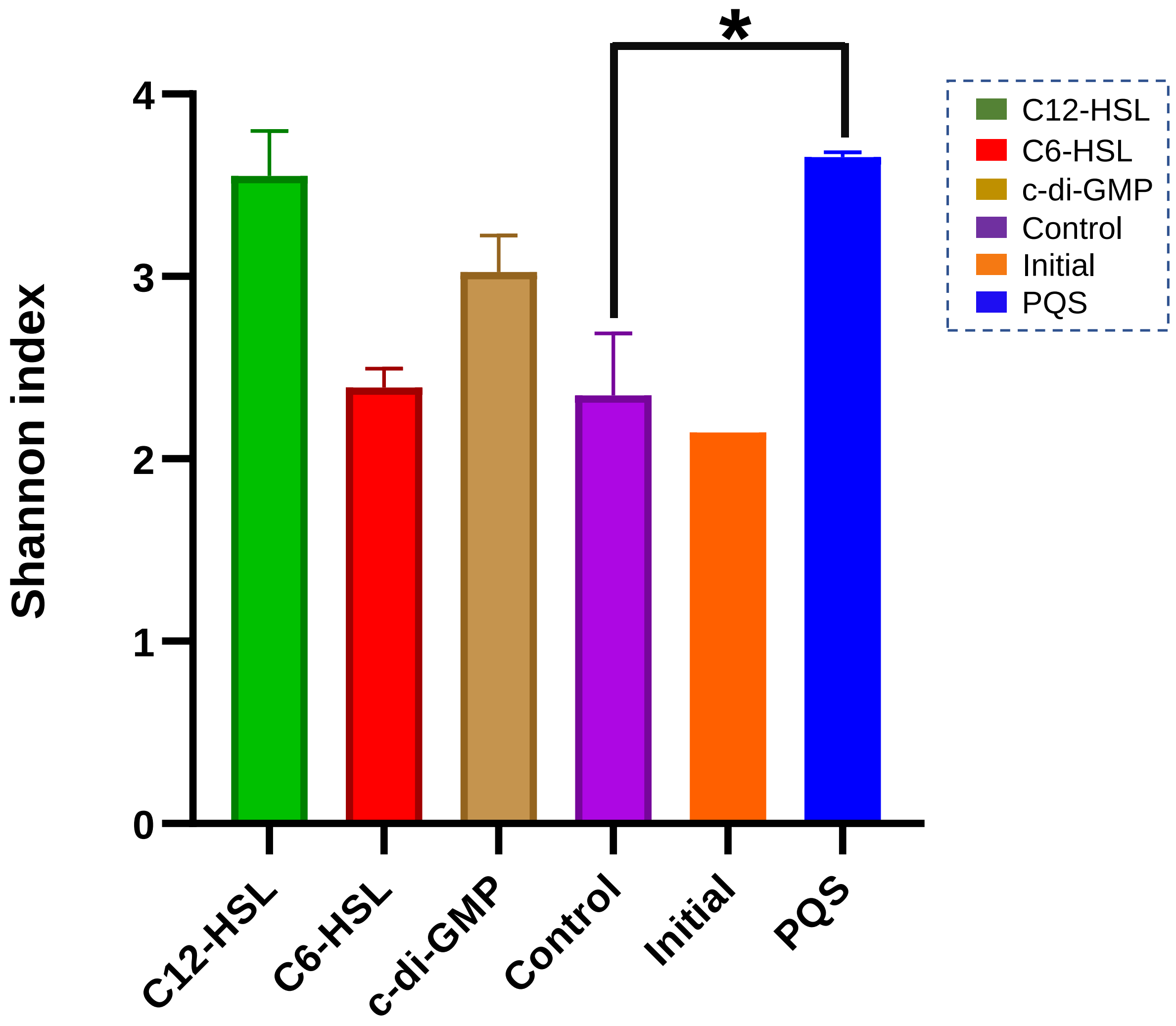

Supplementary Figure S2. Alpha-diversity based on the Shannon index of genus classification in the control and treatment groups (*, p-value <0.05).

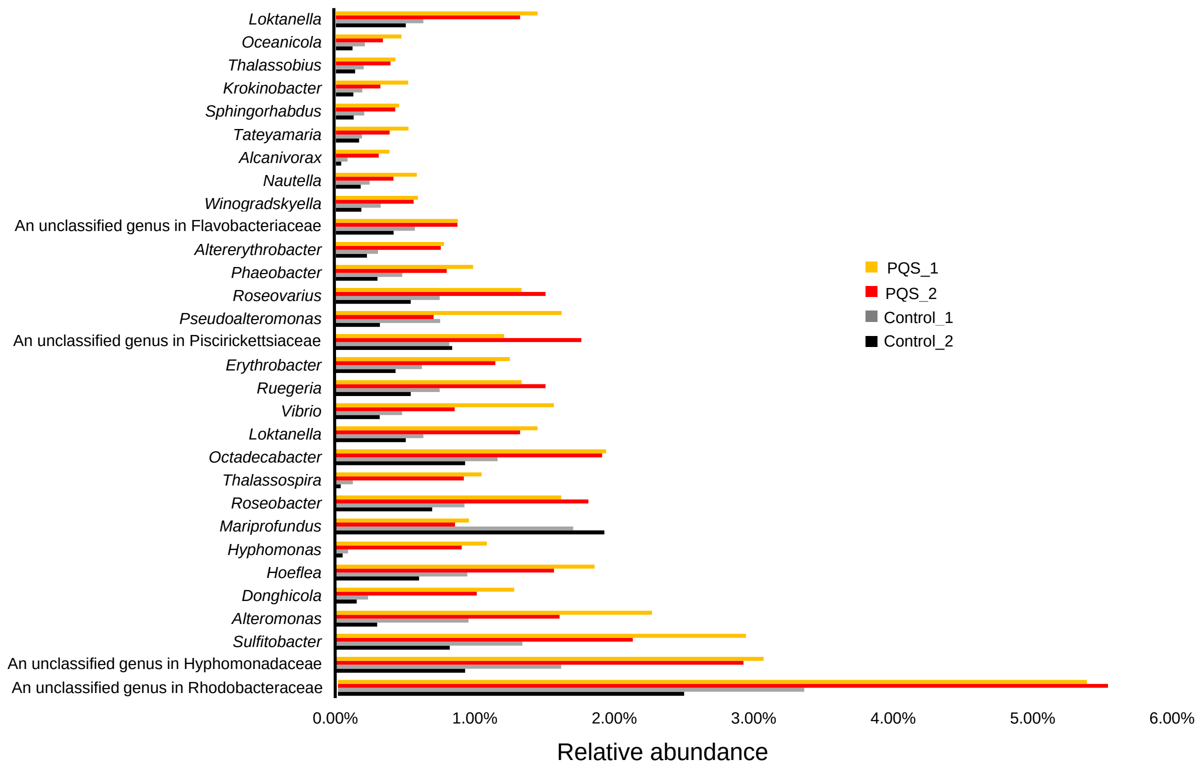

Supplementary Figure S3. SIMPER analysis of the genus composition of the PQS-treated biofilms and the control biofilms in the signal molecule treatment experiment. The top 30 genera based on the contribution to the difference between the PQS-treated biofilms and the control biofilms are shown.

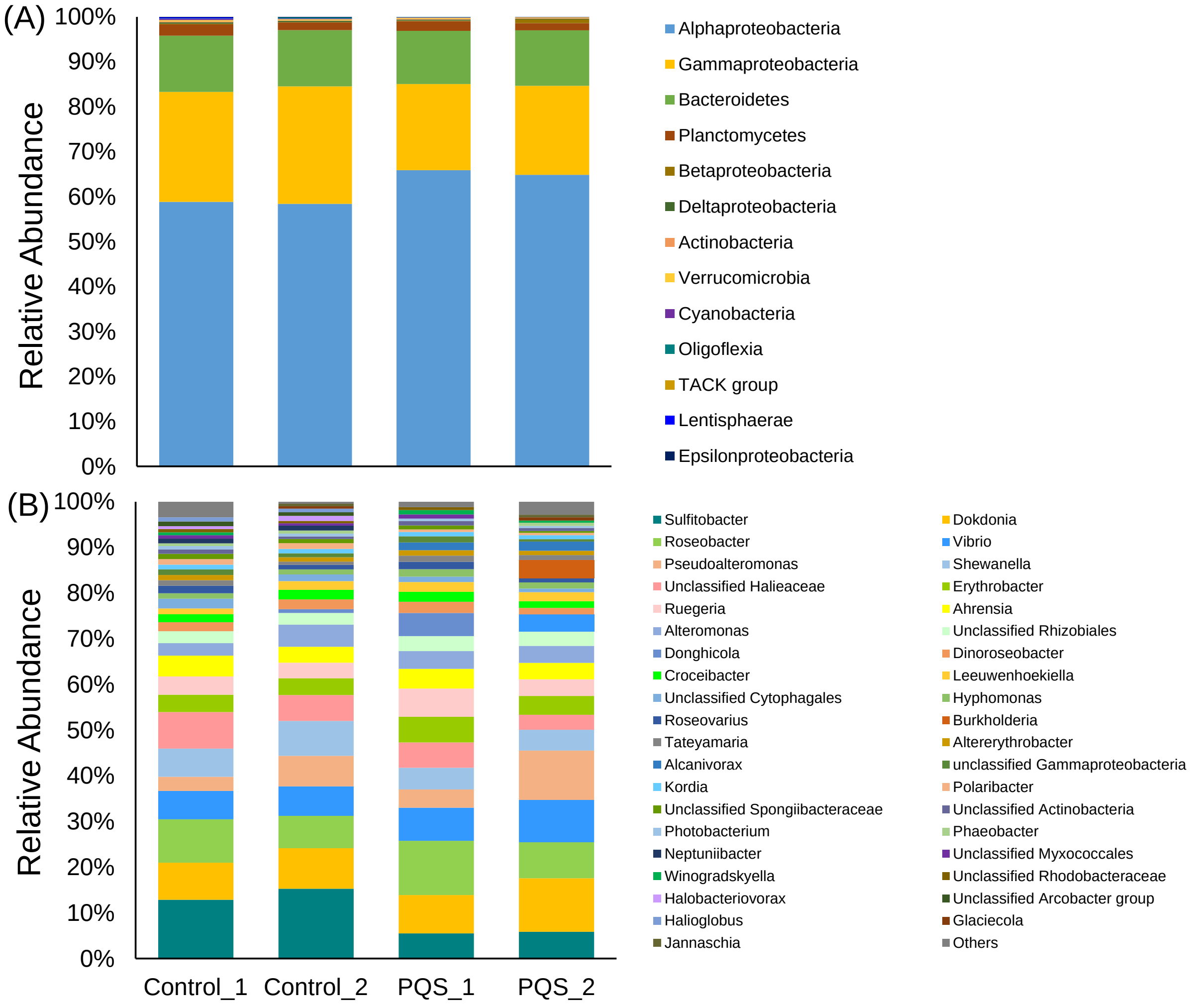

Supplementary Figure S4. Taxonomic affiliation of signal transduction genes in the PQS-treated and the control biofilms. (A) phylum level; (B) genus level.

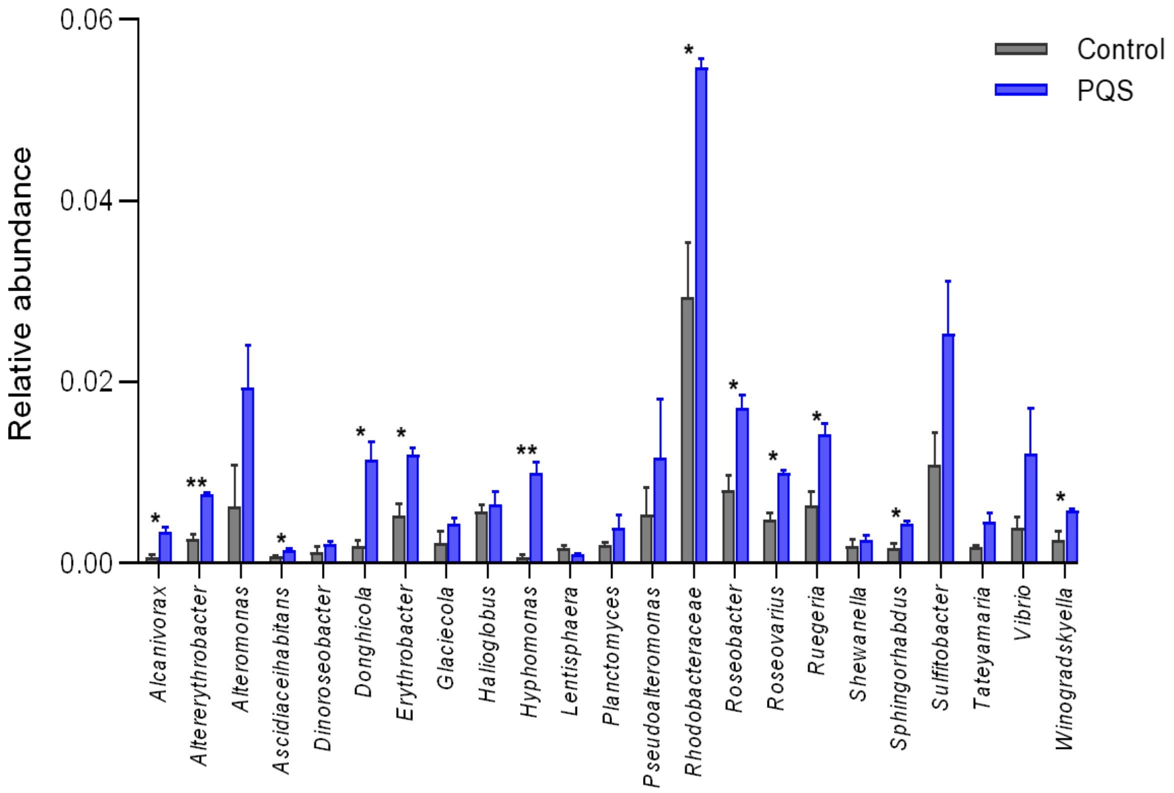

Supplementary Figure S5. Statistical analysis of the genus affiliation of signal transduction genes in the PQS-treated and the control biofilms (*, p-value <0.05; **, p-value <0.01).

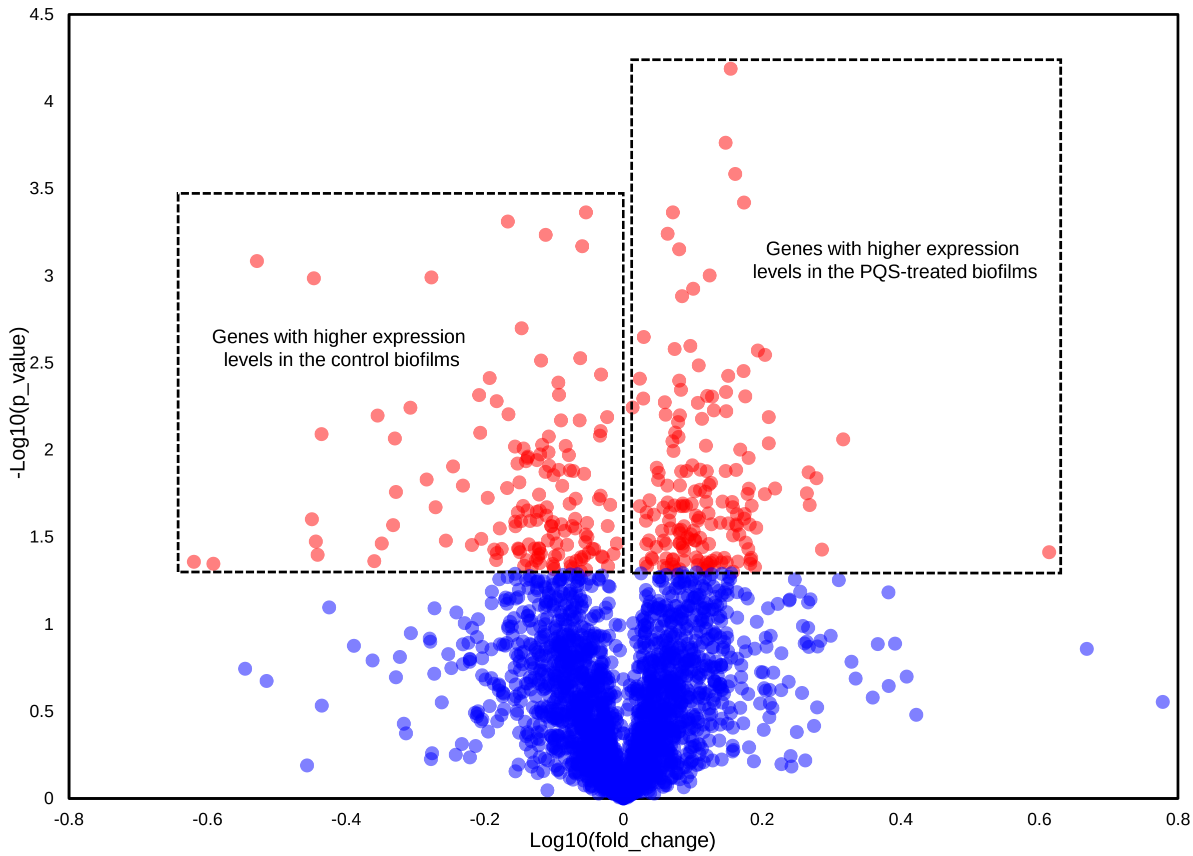

Supplementary Figure S6. Transcriptomic analysis of the *Erythrobacter* sp. HKB8 biofilm treated with PQS. Three biological replicates were performed. The fold change value of a given gene equals to the average TPM in the PQS-treated biofilms divided by the average TPM in the control biofilms (biofilms without treatment). Genes with significant changes (two-tailed Student’s t-test, p-value <0.05) were plotted as the red dots while ones without significant changes were plotted as the blue dots.

Supplementary Table S1. Metagenomic information of the biofilms treated by four different kinds of molecules. Nine-day biofilms were collected from the Hong Kong Water and then subjected to treatment for one day in the laboratory. The biofilm without any treatment was labeled as 'initial', and biofilms treated with only filtered seawater were labeled as 'control'. Data size is the total length of DNA bases, and '×2' indicate that the metagenome has two paired files.

Biofilm metagenomes	Data size (bp)	Sampling locations	Sampling time	Biofilm substrata	Data accession (in NCBI)
Initial	8,687,417,700×2	Port Shelter, Hong Kong (22.34, 114.27)	17th-26th January 2018	Petri dish	Bioproject PRJNA513416
Control_1	8,614,537,150×2	Port Shelter, Hong Kong (22.34, 114.27)	17th-26th January 2018	Petri dish	Bioproject PRJNA513416
Control_2	9,404,574,600×2	Port Shelter, Hong Kong (22.34, 114.27)	17th-26th January 2018	Petri dish	Bioproject PRJNA513416
C6_1	8,348,004,300×2	Port Shelter, Hong Kong (22.34, 114.27)	17th-26th January 2018	Petri dish	Bioproject PRJNA513416
C6_2	9,433,105,650×2	Port Shelter, Hong Kong (22.34, 114.27)	17th-26th January 2018	Petri dish	Bioproject PRJNA513416
C12_1	8,022,271,050×2	Port Shelter, Hong Kong (22.34, 114.27)	17th-26th January 2018	Petri dish	Bioproject PRJNA513416
C12_2	10,214,193,300×2	Port Shelter, Hong Kong (22.34, 114.27)	17th-26th January 2018	Petri dish	Bioproject PRJNA513416
c-di-GMP_1	9,740,672,250×2	Port Shelter, Hong Kong (22.34, 114.27)	17th-26th January 2018	Petri dish	Bioproject PRJNA513416
c-di-GMP_2	10,001,008,350×2	Port Shelter, Hong Kong (22.34, 114.27)	17th-26th January 2018	Petri dish	Bioproject PRJNA513416
PQS_1	7,807,225,350×2	Port Shelter, Hong Kong (22.34, 114.27)	17th-26th January 2018	Petri dish	Bioproject PRJNA513416
PQS_2	8,294,609,300×2	Port Shelter, Hong Kong (22.34, 114.27)	17th-26th January 2018	Petri dish	Bioproject PRJNA513416

Supplementary Table S2. The information of signal molecules used in the laboratory experiment.

Group name	Signal molecules	Significance	Incubation conditions in laboratory	Replicate(s)	Molecule concentration (μM) in filtered seawater	The volume of molecule solution solvent DMSO* (μl)
Initial	—	—	No incubation	1	—	—
Control	—	—	24h, 22°C, stationary	2	—	3
C6	N-Hexanoyl-L-homoserine lactone	Acts as a QS signal molecule in Gram-negative bacteria	24h, 22°C, stationary	2	10	3
C12	N-(3-Oxododecanoyl)-L-homoserine lactone	A QS signal which is involved in regulating diverse virulence determinants including exotoxin A, elastase, alkaline protease and haemolysin	24h, 22°C, stationary	2	10	3
c-di-GMP	Cyclic-di-GMP	A ubiquitous second messenger, control cell adhesion and persistence of multicellular communities	24h, 22°C, stationary	2	2.5	3
PQS	2-Heptyl-3-hydroxy-4(1H)-quinolone	A QS signal which regulates the production of virulence determinants and influences biofilm development	24h, 22°C, stationary	2	10	3

*DMSO: dimethyl sulfoxide

Supplementary Table S3. Genomic information of the *Erythrobacter* sp. HKB8 bacterium. This bacterium was isolated from a Hong Kong biofilm in July of 2018. The completeness and potential contamination were evaluated by CheckM.

Strain name	*Erythrobacter* sp. HKB8
Isolation source	Marine biofilm
Taxonomic classification	Bacteria; Proteobacteria; Alphaproteobacteria; Sphingomonadales; Erythrobacteraceae; Erythrobacter
Genome information	
Number of contigs	1
Genome completeness (%)	99.48
Potential contamination (%)	0
Length of sequence (bp)	2,746,849
GC content (%)	64.41
Number of ORFs	2637
Number of ORFs annotated by KEGG	1858
Genome accession (in NCBI)	Bioproject PRJNA513246

Supplementary Table S4. Information of the transcriptomes of *Erythrobacter* sp. HKB8. Biofilms of this bacterium were treated with PQS and then transcriptomic analysis was performed. Number of clean reads is the total reads number of DNA sequences, and '×2' indicate that the transcriptome has two paired files.

	Number of clean reads	Length of reads (bp)	Data accession (in NCBI)
HKB8-Control_1	4,736,866×2	150	Bioproject PRJNA513396
HKB8-Control_2	4,031,565×2	150	Bioproject PRJNA513396
HKB8-Control_3	4,357,113×2	150	Bioproject PRJNA513396
HKB8-PQS_1	4,123,392×2	150	Bioproject PRJNA513396
HKB8-PQS_2	4,350,951×2	150	Bioproject PRJNA513396
HKB8-PQS_3	4,522,054×2	150	Bioproject PRJNA513396
